# Standardized IMGT® Nomenclature of Salmonidae IGH Genes, the Paradigm of Atlantic Salmon and Rainbow Trout: From Genomics to Repertoires

**DOI:** 10.3389/fimmu.2019.02541

**Published:** 2019-11-12

**Authors:** Susana Magadan, Aleksei Krasnov, Saida Hadi-Saljoqi, Sergey Afanasyev, Stanislas Mondot, Delphine Lallias, Rosario Castro, Irene Salinas, Oriol Sunyer, John Hansen, Ben F. Koop, Marie-Paule Lefranc, Pierre Boudinot

**Affiliations:** ^1^Immunology Laboratory, Biomedical Research Center, University of Vigo, Vigo, Spain; ^2^Department of Biology, Center of Evolutionary and Theoretical Immunology, University of New Mexico, Albuquerque, NM, United States; ^3^Nofima AS, Norwegian Institute of Food, Fisheries and Aquaculture Research, Tromsø, Norway; ^4^IMGT®, The International ImMunoGeneTics Information System® (IMGT), Laboratoire d'ImmunoGénétique Moléculaire (LIGM), Institut de Génétique Humaine (IGH), CNRS, University of Montpellier, Montpellier, France; ^5^Sechenov Institute of Evolutionary Physiology and Biochemistry, Saint Petersburg, Russia; ^6^MICALIS, Institut National de la Recherche Agronomique, Université Paris-Saclay, Jouy-en-Josas, France; ^7^Génétique Animale et Biologie Intégrative (GABI), INRA, AgroParisTech, Université Paris-Saclay, Jouy-en-Josas, France; ^8^Virologie et Immunologie Moléculaires (VIM), Institut National de la Recherche Agronomique (INRA), Université Paris-Saclay, Jouy-en-Josas, France; ^9^Pathobiology Department, School of Veterinary Medicine, University of Pennsylvania, Philadelphia, PA, United States; ^10^Western Fisheries Research Center, U.S. Geological Survey, Seattle, WA, United States; ^11^Department of Biology, University of Victoria, Victoria, BC, Canada

**Keywords:** immunoglobulin, antibody repertoire, salmonid fish, VDJ annotation, comparative immunology

## Abstract

In teleost fish as in mammals, humoral adaptive immunity is based on B lymphocytes expressing highly diverse immunoglobulins (IG). During B cell differentiation, IG loci are subjected to genomic rearrangements of V, D, and J genes, producing a unique antigen receptor expressed on the surface of each lymphocyte. During the course of an immune response to infections or immunizations, B cell clones specific of epitopes from the immunogen are expanded and activated, leading to production of specific antibodies. Among teleost fish, salmonids comprise key species for aquaculture. Rainbow trout (*Oncorhynchus mykiss*) and Atlantic salmon (*Salmo salar*) are especially important from a commercial point of view and have emerged as critical models for fish immunology. The growing interest to capture accurate and comprehensive antibody responses against common pathogens and vaccines has resulted in recent efforts to sequence the IG repertoire in these species. In this context, a unified and standardized nomenclature of salmonid IG heavy chain (IGH) genes is urgently required, to improve accuracy of annotation of adaptive immune receptor repertoire dataset generated by high-throughput sequencing (AIRRseq) and facilitate comparisons between studies and species. Interestingly, the assembly of salmonids IGH genomic sequences is challenging due to the presence of two large size duplicated IGH loci and high numbers of IG genes and pseudogenes. We used data available for Atlantic salmon to establish an IMGT standardized nomenclature of IGH genes in this species and then applied the IMGT rules to the rainbow trout IGH loci to set up a nomenclature, which takes into account the specificities of Salmonid loci. This unique, consistent nomenclature for Salmonid IGH genes was then used to construct IMGT sequence reference directories allowing accurate annotation of AIRRseq data. The complex issues raised by the genetic diversity of salmon and trout strains are discussed in the context of IG repertoire annotation.

## Introduction

Vertebrate species with jaws (*Gnasthostomata*) that appeared more than 400 million years ago are all characterized by an adaptive immune system based on B and T cells along with the huge diversity and specificity of their antigen receptors, the immunoglobulins (IG) or antibodies and the T cell receptors (TR), respectively (1, 2). The analysis of the germline IGH locus defines the genomic repertoire with the identification of the functional variable (V), diversity (D), and joining (J) genes that participate in the synthesis of VH domains. It also allows the identification of the functional constant (C) genes that encode the constant regions of the heavy chains and define their isotypes (3–7).

In teleost fish, B cell clonal responses are induced by infection or immunization, as described in humans or mice. Antibodies constitute a key factor for fish specific immunity and for the protection afforded by vaccines. As key species in aquaculture, Salmonids (family Salmonidae) including rainbow trout (*Oncorhynchus mykiss; Oncmyk*) and Atlantic salmon (*Salmo salar; Salsal*) constitute important models for the study of antibodies and B cell responses in fish.

Several groups started to clone and sequence IGH cDNA from rainbow trout in the early 1990s (8–12). Comparison of VH domains (V-D-J-REGION) expressed in trout stocks from Sweden, France, and the US revealed differences in IGHV subgroup usage: subgroups named 8, 9, 10, and 11 were found only in Swedish stocks while subgroups 4 and 7 were only found in French stocks and subgroup 5 (now part of IGHV1) was found in Swedish, French, and US stocks. These observations suggested genetic differences between the IGHV gene germline repertoires of different populations, but this was not fully clear due to the very small numbers of sampled individuals. In 1996, expressed VH domain sequences were classified into a set of 11 IGHV subgroups, defining a first unified nomenclature for rainbow trout (13). A more extensive study performed in 2006 on American trout by the group of Steve Kaattari found all these subgroups expressed, indicating that IGHV subgroups may have a wider distribution than previously suggested. Two additional subgroups expressed at low frequency were also discovered in this survey (14), leading to a repertoire of 13 IGHV subgroups. These subgroups were used for an IMGT gene table created in 2009, with a provisional gene nomenclature (letter S) for rainbow trout IGHV [path to access: IMGT Repertoire (IG and TR) >1. Locus and genes > Gene tables > IGHV > Rainbow trout (*O. mykiss*)]^1^.

In Atlantic salmon, Solem et al. described in 2001 nine IGHV subgroups (15), seven of which corresponded to IGHV subgroups defined in rainbow trout (1, 2, 3, 6, 8, 9, and 11). Southern blot experiments suggested that the number of genes per subgroup could vary between 1 and 7 ± 10. This work also clearly established that Atlantic salmon IGHV genes were rearranged and transcribed from both of the two Atlantic salmon IGH loci (IGH locus A on chromosome 6 and IGH locus B on chromosome 3), which were most likely produced by the salmonid whole genome duplication. These data actually suggested that genes from some subgroups could be expressed only from a single locus, while genes from other subgroups were expressed from both A and B loci. This analysis was later extended and refined in 2010 by Yasuike et al. from a complete assembly of the Atlantic salmon IGH A and B loci based on sequences of 24 bacterial artificial chromosomes (BAC) (16). This study provided a first map of the organization of the duplicated IGH loci of a salmonid species. Ninety-nine IGHV genes were found in locus A, and 103 in locus B; 23 IGHV genes are functional in locus A, and 32 in locus B. Using the IMGT threshold of 75% identity for the V-REGION, 18 IGHV subgroups were defined in this work (16). Subgroups that did fit with the IGHV subgroups established in rainbow trout were given a subgroup number consistent with the online 2009 IMGT gene table [IMGT Repertoire (IG and TR) > 1. Locus and genes > Gene tables > IGHV > Atlantic salmon (*Salmo salar*)]^1^.

As new genome assemblies of Atlantic salmon and rainbow trout have been recently made available, we decided to annotate the IGH locus of these species and to establish a common nomenclature of IGH genes based on IMGT rules. We used data previously published for Atlantic salmon (16) to develop a prototype for the Salmonid IMGT standardized nomenclature. We also applied the IMGT rules to the rainbow trout IGH loci as a novel example of IMGT genomic annotation. The objective was to take into account the specificities of the Salmonid loci and to develop a unique, consistent nomenclature, while respecting the IMGT Scientific chart rules and standards. These standards are based on the concepts of identification (keywords), classification (gene and allele nomenclature), description (labels), and numbering (IMGT unique numbering and IMGT Collier de Perles) (3). It is important to note that a consistent nomenclature is crucial to build IMGT reference directory sets that are constituted by the V-REGION, D-REGION, and J-REGION of each IMGT reference allele from IMGT/LIGM-DB (same accession numbers as GenBank, ENA, and DDBJ) (17). These reference directory sets are the fundamental basis for annotation of repertoire datasets produced by high-throughput AIRRseq approaches for the analysis of expressed repertoires, in particular to define expressed clonotypes (18–20). The IMGT reference directories are built following the classification of the V, D, J, and C genes and alleles according to the IMGT rules and the assignment of the IMGT functionality: functional (F), open reading frame (ORF), or pseudogene (P) (IMGT Scientific chart > IMGT functionality)^1^ (3). These rules ensure that the nomenclature is consistent within and between species, and can be updated when more sequence data become available. Reference directory sets are used by IMGT/V-QUEST and IMGT/JunctionAnalysis (21, 22) for detailed analysis of nucleotide (nt) sequences of V domains [V-(D)-J-REGION]; by IMGT/DomainGapAlign, which provides alignments of amino acid (AA) sequences with the closest V and J regions for V domains and the closest C exons for C domains (23); by IMGT Collier de Perles based on the IMGT unique numbering for V and C domains (24, 25); and by IMGT/HighV-QUEST (26, 27) for high-throughput sequence analysis of expressed IGH repertoires and clonotype definition (18–20). Importantly, IMGT reference directory sets are freely available for the academic community and can be used by other programs developed for repertoire analysis.

In this work, we produced reference directory sets for IGH loci of Atlantic salmon and rainbow trout, based on a unique nomenclature developed for salmonids and following IMGT rules. We show how the particularities of salmonid IGH loci (duplicated loci in each haplotype, large number of genes and pseudogenes) were taken into account and how reference directory sets can be used for annotation of IGH expression datasets. We also discuss how the nomenclature and reference directories can be updated with new data and extended to other salmonid species.

## Materials and Methods

GU129139 and GU129140 from GenBank, ENA, and DDBJ, entered in IMGT/LIGM-DB (Rel. 201839-1) and IMGT annotated (GU129139 in Rel. 201923-5, Last updated, Version 11 and GU129140 in Rel. 201930-1, Last updated, Version 10), were selected as *S. salar* (*Salsal*) IMGT IGH locus prototypes. Sequences from these entries are from Atlantic salmon BAC library (CHORI-214), constructed from a Norwegian aquaculture strain male, from BACPAC Resources, Children's Hospital Oakland Research Institute (CHORI) (16). GU129139 (931200 bp) (Salsal locus A, ssa06, IMGT locus ID: Salsal_IGH_1) is in reverse (REV) orientation on chromosome 6 whereas GU129140 (1063283 bp) (Salsal IGH locus B, ssa03, IMGT locus ID: Salsal_IGH_2) is in forward (FWD) orientation on chromosome 3.

For obtaining IMGT gene names, newly identified Atlantic salmon and rainbow trout IGH genes and alleles from genome assemblies were submitted to the IG, T cell receptors (TR), and major histocompatibility (MH) Nomenclature Sub-Committee (IMGT-NC) of the International Union of Immunological Societies (IUIS) Nomenclature Committee^2^^,^^3^. Two IMGT_NC reports #2019-5-0131 and #2019-7-0220^2^ comprise the submission of 75 Atlantic salmon IGHV sequences from two accession numbers NC_027305.1 and NC_027302.1. These reports concern 75 different genes [35 Atlantic salmon IGHV on NC_027305.1 (Salsal locus A, ssa06) and 40 Atlantic salmon IGHV genes on NC_027302.1 (Salsal locus B, ssa03)] and correspond to 75 new alleles (61 of them are ^*^01 and 14 are ^*^02).

Two new entries were created in IMGT/LIGM-DB: IMGT000028 for Salsal locus A [*S. salar* (Atlantic salmon), taxon:8030, breed: double haploid, assembly GCF_000233375.1, GenBank assembly ID: GCA_000233375.4, chromosome 6, CM003284.1 (20520824–22238370, complement), IGH locus A] [this entry includes IMGT annotated genes from NC_027305.1 (Salsal ssa06)] and IMGT000029 for Salsal locus B [*S. salar* (Atlantic salmon), taxon:8030, breed: double haploid, assembly GCF_000233375.1, GenBank assembly ID: GCA_000233375.4, chromosome 3, CM003281.1 (77578187–79383607), IGH locus B] [this entry includes IMGT annotated genes from NC_027302.1 (Salsal ssa03)].

The rainbow trout genome (assembly: Omyk_1.0, June 2017; GenBank assembly accession GCA_002163495.1) obtained from the homozygous Swanson clonal line was examined to locate IGH locus. Two IGH loci were identified, locus A on chromosome 13 (Oncmyk chr13) and locus B on chromosome 12 (Oncmyk chr12), both of them are in forward (FWD) orientation. The IMGT-NC Report #2019-10-040^2^ comprises the submission of 181 rainbow trout IGH gene sequences from NC_035089.1 (Oncmyk Omy13) and NC_035088.1 (Oncmyk Omy12). This IMGT-NC report concerns 181 different genes: 74 genes in locus A on Oncmyk chr 13 (49 IGHV, 11 IGHD, 10 IGHJ, and 4 IGHC on NC_035089.1) and 107 genes in locus B on Oncmyk chr 12 (80 IGHV, 13 IGHD, 9 IGHJ, and 5 IGHC on NC_035088.1) and corresponds to 181 new alleles ^*^01. Two new entries were created in IMGT/LIGM-DB: IMGT000043 (IMGT/LIGM-DB) for Oncmyk locus A [*O. mykiss* (rainbow trout), taxon:8022, isolate: Swanson, assembly Omyk_1.0, GenBank assembly ID: GCF_002163495.1, chromosome 13: CM007947.1 (48012355–48422510), IGH locus A] [this entry includes IMGT annotated genes from NC_035089.1 (Oncmyk Omy13)] and IMGT000044 for Oncmyk locus B [*O. mykiss* (rainbow trout), taxon:8022, isolate: Swanson, assembly Omyk_1.0, GenBank assembly ID: GCF_002163495.1, chromosome 12: CM007946.1 (81302817–81805590), IGH locus B] [this entry includes IMGT annotated genes from NC_035088.1 (Oncmyk Omy12)].

## Results

The complete and correct assembly of the Salmonidae IGH loci is a significant challenge owing to (i) the existence of two duplicated loci due to the tetraploidization (named locus A and locus B), (ii) the large size of each locus, (iii) the high number of different IGHV subgroups compared to mammals, (iv) the internal amplification and potential gene conversion that occurred inside each locus during their evolution, and (v) the very high number of pseudogenes, many of them partial, relative to the functional genes.

We therefore explored how the standardized IMGT nomenclature could allow the identification and classification of genes and alleles in incomplete or not yet fully annotated genome assemblies. The IGH data published for Atlantic salmon (16), largely based on BAC sequencing, were used as a prototype for establishing the standardized IMGT nomenclature for salmonids and for dealing, by comparison, with newly identified IGH genes from both Atlantic salmon and rainbow trout genome assemblies. The particularities of these IGH loci (in particular the tetraploidization) were taken into consideration for consistency between salmonid species.

### From IG Classes to IMGT Constant (C) Gene Names

Three antibody classes have been identified in fish, namely, IgM, IgD, and IgT, while IgG, IgA, and IgE are absent (28). IgM and IgD are generally co-expressed at the cell surface of the same B cells through alternative splicing, as in mammals. Soluble IgM are tetrameric and constitute the main antibody class in serum. A third class, IgT, is expressed in most fish groups including salmonids. Interestingly, the IG-Heavy-Tau chains of IgT have a VH domain that results from independent V-D-J rearrangements, and is not obtained by a switch process (29). IgT has been found only in bony fish and is particularly involved in mucosal immunity and protection (30). IGHD was cloned and characterized in rainbow trout and Atlantic salmon, in parallel to the discovery of IGHT encoding the third fish IG-Heavy-Tau isotype (28, 29) and then in Atlantic salmon (31).

By convention, IMGT groups are designated by the locus and gene type. Based on the four gene types, V (variable), D (diversity), J (joining), and C (constant), the IGH genes belong to four groups: IGHV, IGHD, IGHJ, and IGHC. For the IGH locus, the constant genes are designated by the letter (and, if relevant, number) corresponding to the encoded isotype (IGHT, IGHM, and IGHD), instead of using the letter C.

The salmonid IGHC genes belong to three subgroups IGHM, IGHD, and IGHT and encode, when functional, the C-REGION of the heavy chain defining these three isotypes, IG-Heavy-Mu (heavy chain of the IgM class), IG-Heavy-Delta (heavy chain of the IgD class), and IG-Heavy-Tau (heavy chain of the IgT class) (Table 1). Salmonid locus A and locus B were assigned based on the literature, with the letter D (for “duplicated”) added to the conventional gene names for locus B.

**Table 1:** Salmonid IG receptor classes, heavy chain types, and IGHC gene names.

**IG receptor class**	**IG heavy chain type**	**IG C-gene group**	**IG C-gene subgroup**	**IGHC gene names**			
				***Salmo salar***		***Oncorhynchus mykiss***	
				**Locus A**	**Locus B**	**Locus A**	**Locus B**
IgM	IG-Heavy-Mu	IGHC	IGHM	IGHM	IGHMD	IGHM	IGHMD
IgD	IG-Heavy-Delta	IGHC	IGHD	IGHD	IGHDD	IGHD	IGHDD
IgT	IG-Heavy-Tau	IGHC	IGHT	IGHT1 IGHT2 IGHT3 IGHT4 IGHT5	IGHT1D IGHT2D IGHT3D	IGHT1 IGHT2	IGHT1D

### Atlantic Salmon IGH Constant Genes and Associated D and J Genes

**Figure 1:**
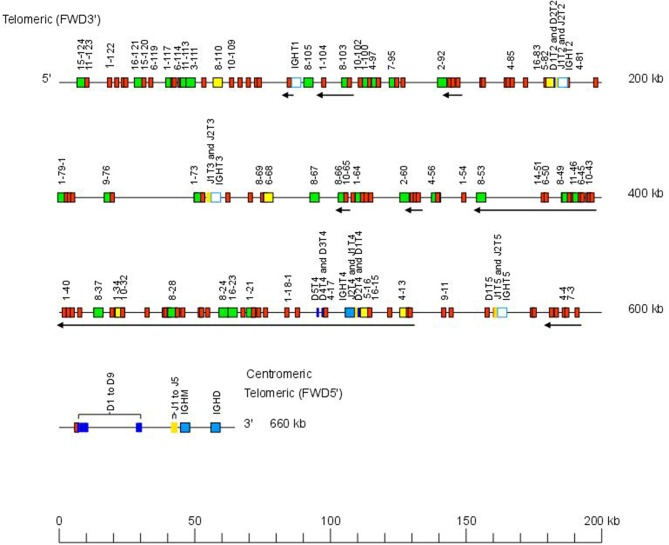
Organization of the Atlantic salmon (*Salmo salar*) locus A. The orientation of the Atlantic salmon (*S. salar*) IGH locus on chromosome 6 (locus A—Salsal ssa06) is reverse (REV). IGH gene names are according to IMGT nomenclature (3). Single arrows show genes whose polarity is opposite to that of the D-J-C CLUSTER comprising IGHD (D1–D9)-IGHJ (J1–J5)-IGHM-IGHD. Gene names of IGHV pseudogenes with frameshift(s) in the V-REGION are not displayed due to lack of space in the locus map at the IMGT standardized scale (line = 200 kb). All gene names are displayed in a zoom (line = 100 kb) on the IMGT site [IMGT Repertoire (IG and TR) > Locus and genes > Locus representations]^1^. Color coding of genes is according to the IMGT Color menu for genes (IMGT Scientific chart > 4. Representation Rules > IMGT color menu > 11. Color menu for genes)^1^: V-GENE (green: functional, yellow: ORF, red: pseudogene), D-GENE (blue: functional), J-GENE (yellow: functional), and C-GENE (blue: functional, white box: pseudogene). With permission of IMGT^®^, the international ImMunoGenetics information system^®^.

**Figure 2:**
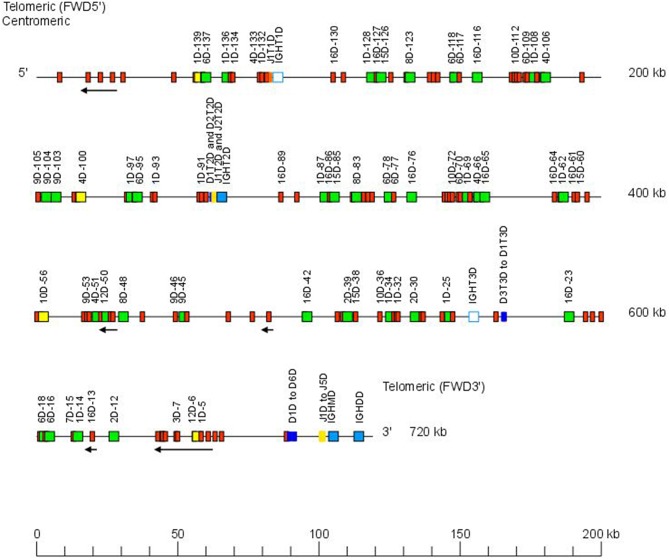
Organization of the Atlantic salmon (*S. salar*) locus B. The orientation of the Atlantic salmon (*S. salar*) IGH locus on chromosome 3 (locus B—Salsal ssa03) is forward (FWD). IGH gene names are according to IMGT nomenclature (3). Single arrows show genes whose polarity is opposite to that of the D-J-C CLUSTER comprising IGHD (D1–D9)-IGHJ (J1–J5)-IGHM-IGHD. Gene names of IGHV pseudogenes with frameshift(s) in the V-REGION are not displayed due to lack of space in the locus map at the IMGT standardized scale (line = 200 kb). All gene names are displayed in a zoom (line = 100 kb) on the IMGT site [IMGT Repertoire (IG and TR) > Locus and genes > Locus representations]^1^. Color coding of genes is according to the IMGT Color menu for genes (IMGT Scientific chart > 4. Representation Rules > IMGT color menu > 11. Color menu for genes)^1^: V-GENE (green: functional, yellow: ORF, red: pseudogene), D-GENE (blue: functional), J-GENE (yellow: functional), and C-GENE (blue: functional, white box: pseudogene). With permission of IMGT^®^, the international ImMunoGenetics information system^®^.

The Atlantic salmon IGH locus A, which is in a reverse (REV) orientation on chromosome 6 and spans 660 kilobases (kb) (with the V genes encompassing 600 kb) (Figure 1) includes 7 IGHC genes with 17 associated IGHD genes and 13 IGHJ genes. The Atlantic salmon IGH locus B, which is in forward (FWD) orientation on chromosome 3 and spans 720 kb (with the V genes encompassing 670 kb) (Figure 2) includes 5 IGHC genes with 11 associated IGHD genes and 8 IGHJ genes. The constant region of the IG-Heavy-Mu chain and of the IG-Heavy-Delta are encoded by a unique gene per locus (IGHM and IGHD for locus A and IGHMD and IGHDD for locus B) preceded by a D-J cluster. There are several IG-Heavy-Tau genes (IGHT), but the associated D-J cluster may be incomplete (lacking D and/or J genes). In Atlantic salmon, there is only one IGHT functional (F) gene per locus, IGHT4 for locus A and IGHT2D for locus B, each one having a complete D-J cluster (Table 2).

**Table 2:** Atlantic salmon (*Salmo salar*) IGH constant C genes and associated D and J genes.

***Salmo salar*** **locus A** **on chromosome 6 (Salsal ssa06)**						***Salmo salar*** **locus B** **on chromosome 3 (Salsal ssa03)**					
**IGHD genes**		**IGHJ genes**		**IGHC** **genes**		**IGHD genes**		**IGHJ genes**		**IGHC genes**	
–		–		IGHT1	P	–		IGHJ1T1D	P	IGHT1D	P
IGHD1T2	F	IGHJ1T2	F	IGHT2	P	IGHD1T2D	F	IGHJ1T2D	F	IGHT2D	F
IGHD2T2	F	IGHJ2T2	F			IGHD2T2D	F	IGHJ2T2D	F		
		IGHJ1T3	F	IGHT3	P	IGHD1T3D	F			IGHT3D	P
		IGHJ2T3	F			IGHD2T3D	F				
IGHD1T4	F	IGHJIT4	F	IGHT4	F	IGHD3T3D	F				
IGHD2T4	F	IGHJ2T4	F								
IGHD3T4	F										
IGHD4T4	F										
IGHD5T4	F										
IGHD1T5	F	IGHJIT5	P	IGHT5	P						
		IGHJ2T5	F								
IGHD1	F	IGHJ1	F	IGHM	F	IGHD1D	F	IGHJID	F	IGHMD	F
IGHD2	F	IGHJ2	ORF			IGHD2D	F	IGHJ2D	F		
IGHD3	F	IGHJ3	F			IGHD3D	F	IGHJ3D	F		
IGHD4	F	IGHJ4	F			IGHD4D	F	IGHJ4D	F		
IGHD5	F	IGHJ5	F, ORF			IGHD5D	F	IGHJ5D	F, ORF		
IGHD6	F					IGHD6D	F				
IGHD7	F										
IGHD8	F										
IGHD9	F										
				IGHD	F					IGHDD	F

*F, functional; ORF, open reading frame; P, pseudogene. The functionality is according to IMGT functionality (IMGT Scientific chart > IMGT functionality)^1^ (3)*.

In the Atlantic salmon locus A, the D and J genes associated to IGHT genes comprise two D (IGHD1T2 and IGHD2T2) and two J (IGHJ1T2 and IGHJ2T2) upstream of the pseudogene (P) IGHT2, two J (IGHJ1T3 and IGHJ2T3) upstream of IGHT3 (P), five D (IGHD1T4 to IGHD5T4) and two J (IGHJ1T4 and IGHJ2T4) genes, all of them functional, upstream of IGHT4 (F) and one D (IGHD1T5) and two J (IGHJ1T5 and IGHJ2T5) upstream of IGHT5 (P). There is no IGHD or IGHJ upstream of IGHT1 (P) (Table 2). The D and J associated to IGHM and IGHD comprise nine D (IGHD1 to IGHD9), all of them functional and five J genes, three of them functional (IGHJ1, IGHJ3, and IGHJ4), one with ORF, the IGHJ2, and one with alleles F or ORF (IGHJ5). They are located upstream of IGHM (F) and shared with the IGHD constant gene (F) (Table 2 and Figures S1, S2). Eleven IGHD not directly associated to constant genes are dispersed in locus A (IGHD-1 to IGHD-11).

In the Atlantic salmon locus B, the D and J genes associated to IGHT genes comprise one J (IGHJ1T1D) upstream of IGHT1D (P), two D (IGHD1T2D and IGHD2T2D), and two J (IGHJ1T2D and IGHJ2T2D) all functional upstream of IGHT2D (F) and three D (IGHD1T3D, IGHD2T3D, and IGHD3T3D) downstream of IGHT3D (P) (Table 2 and Figures S1, S2). The D and J genes associated to IGHMD and IGHDD comprise six D (IGHD1D to IGHD6D, all functional) and five J genes (four functional, IGHJ1D to IGHJ4D) and one with alleles F or ORF (IGHJ5D). They are located upstream of IGHMD (F) and shared with the IGHDD constant gene (F) (Table 2 and Figures S1, S2). Six IGHD not directly associated to constant genes are dispersed in locus B (IGHD-1D to IGHD-6D).

IGHD, IGHJ, and IGHC genes are reported in IMGT Gene tables [IMGT Repertoire (IG and TR) > 1. Locus and genes > Gene tables > IGHD > Atlantic salmon (*S. salar*); ibid., IGHJ > Atlantic salmon (*S. salar*); ibid., IGHC > Atlantic salmon (*S. salar*)]^1^.

### Rainbow Trout IGH Constant Genes and Associated D and J Genes

Similar to the Atlantic salmon, the rainbow trout has one functional gene per IGH locus encoding the constant region of the IG-Heavy-Mu (IGHM gene in locus A and IGHMD gene in locus B), the constant region of the IG-Heavy-Delta (IGHD gene in locus A and IGHDD gene in locus B), and the constant region of the IG-Heavy-Tau (IGHT2 gene in locus A and IGHT1D gene in locus B).

The rainbow trout IGH locus A, which spans 360 kb and is in a forward (FWD) orientation on chromosome 13, includes 11 IGHD genes, 10 IGHJ genes, and 4 IGHC genes (Table 3). There are three D and two J genes upstream of IGHT1 (P), two D and two J genes upstream of IGHT2 (F), and six D and six J genes (all of them F) upstream of IGHM (F) and shared with the IGHD (F) constant gene (Figures S1, S2).

**Table 3:** Rainbow trout (*Oncorhynchus mykiss*) IGH constant C genes and associated D and J genes.

***Oncorhynchus mykiss*** **locus A** **on chromosome 13 (Oncmyk Omy13)**						***Oncorhynchus mykiss*** **locus B** **on chromosome 12 (Oncmyk Omy12)**					
**IGHD genes**		**IGHJ genes**		**IGHC** **genes**		**IGHD genes**		**IGHJ genes**		**IGHC genes**	
IGHD1T1	F	IGHJ1T1	F	IGHT1	P	IGHD1T1D	F	IGHJ1T1D	F	IGHT1D	F
IGHD2T1	F	IGHJ2T1	F			IGHD2T1D	F	IGHJ2T1D	F		
IGHD3T1	F					IGHD3T1D	ORF				
						IGHD4T1D	F				
IGHD1T2	F	IGHJ1T2	F	IGHT2	F						
IGHD2T2	F	IGHJ2T2	F								
IGHD1	F	IGHJ1	F	IGHM	F	IGHD1D	F	IGHJ1D	F	IGHMD	
IGHD2	F	IGHJ2	F			IGHD2D	F	IGHJ2D	F		
IGHD3	F	IGHJ3	F			IGHD3D	F	IGHJ3D	F		
IGHD4	F	IGHJ4	F			IGHD4D	F	IGHJ4D	F		
IGHD5	F	IGHJ5	F			IGHD5D	F	IGHJ5D	F		
IGHD6	F	IGHJ6	F			IGHD6D	F	IGHJ6D	F		
								IGHJ7D	F		
				IGHD	F					IGHDD	F

*F, functional; ORF, open reading frame; P, pseudogene. The functionality is according to IMGT functionality (IMGT Scientific chart > IMGT functionality)^1^ (3)*.

The rainbow trout IGH locus B, which spans 485 kb and is in a forward (FWD) orientation on chromosome 12, includes 13 IGHD genes, 9 IGHJ genes, and 3 IGHC genes (Table 3). There are four D genes (1 ORF and 3 F) and two J genes (both F) upstream of IGHT1D (F), and six D and seven J genes (all of them F) upstream of IGHMD (F) and shared with the IGHDD (F) constant gene (Figures S1, S2).

Sequences of rainbow trout IGHD and IGHJ genes and alleles are available in the downloadable IMGT reference directory sets from IMGT/GENE-DB (/download/GENE-DB)^1^ and from IMGT/V-QUEST (/download/V-QUEST/IMGT_V-QUEST_reference_directory/Oncorhynchus_mykiss/IG/IGHD.fasta; ibid., /IGHJ.fasta)^1^. IGHD and IGHJ genes and alleles are reported in the IMGT Gene tables [IMGT Repertoire (IG and TR) > 1. Locus and genes > Gene tables > IGHD > Rainbow trout (*O. mykiss*); ibid., IGHJ > Rainbow trout (*O. mykiss*)]^1^.

The demonstration that there is only one rainbow trout IG-Heavy-Delta complete gene per locus, IGHD in locus A and IGHDD in locus B, respectively, and that these two genes are functional, results from the analysis derived from applying the nomenclature of the Atlantic salmon IGH loci as well as the interpretation of expression data and published references (15, 16, 29, 31). The anomalies (partial IGHD and IGHDD genes with exons in aberrant localizations or in reverse-complementary orientation) are likely artifacts of the current genome assembly. For that reason, the functionality of the IGHD and IGHDD, deduced from literature data and supported by sequences external to the genome assembly, is shown in parentheses in Table 3.

### Atlantic Salmon IGH Variable Genes

The Atlantic salmon IGH locus comprises a total of 303 IGH variable (IGHV) genes (145 IGHV in locus A on Salsal chromosome 6, spanning 600 kb, and 158 IGHV in locus B on Salsal chromosome 3, spanning 670 kb) (Figures 1, 2). There are a total of 67–69 functional genes, 12 ORF, and 222–224 pseudogenes (Table 4).

**Table 4:** Atlantic salmon IGH variable genes.

**IMGT group**	**IMGT subgroup**	**Locus A on Salsal chromosome 6**				**Locus B on Salsal chromosome 3**				**Locus A** **+** **Locus B**			
		**Functional**	**ORF**	**Pseudogene**	**Total**	**Functional**	**ORF**	**Pseudogene**	**Total**	**Functional**	**ORF**	**Pseudogene**	**Total**
IGHV	IGHV1	7	1	24	32	12	2	23	37	19	3	47	69
	IGHV2	2	0	0	2	2(+1)^*^	0	5(+1)^*^	8	4(+1)^*^	0	5(+1)^*^	10
	IGHV3	1	0	4	5	1	0	5	6	2	0	9	11
	IGHV4	2	2	12	16	3	1	14	18	5	3	26	34
	IGHV5	0	2	2	4	0	0	4	4	0	2	6	8
	IGHV6	1	1	16	18	6	0	20	26	7	1	36	44
	IGHV7	1	0	2	3	0	0	3	3	1	0	5	6
	IGHV8	10(+1)^*^	1	9(+1)^*^	21	3	0	5	8	13(+1)^*^	1	14(+1)^*^	29
	IGHV9	1	0	4	5	3	0	5	8	4	0	9	13
	IGHV10	0	0	14	14	0	1	8	9	0	1	22	23
	IGHV11	2	0	6	8	0	0	6	6	2	0	12	14
	IGHV12	0	0	1	1	1	1	0	2	1	1	1	3
	IGHV13	0	0	1	1	0	0	2	2	0	0	3	3
	IGHV14	0	0	1	1	0	0	1	1	0	0	2	2
	IGHV15	1	0	5	6	2	0	3	5	3	0	8	11
	IGHV16	1	0	7	8	5	0	10	15	6	0	17	23
Total		29(+1)^*^	7	108(+1)^*^	145	38(+1)^*^	5	114(+1)^*^	158	67(+2)^*^	12	222(+2)^*^	303

*Number of IGHV genes are given per subgroup and per locus A or B, and per IMGT functionality (functional, ORF, pseudogene) (3)*.

* *An asterisk indicates that the following genes have alleles with different functionalities: Functional or Pseudogene (IGHV2D-12 and IGHV8-58)*.

Based on the percentage of identity between nucleotide sequences of the V-REGION (threshold 75%), the Atlantic salmon 303 IGHV genes can be classified into 16 IGHV subgroups. IGHV genes are reported in IMGT Gene tables [IMGT Repertoire (IG and TR) > 1. Locus and genes > Gene tables > IGHV > Atlantic salmon (*S. salar*)]^1^. Correspondence with previous gene names is indicated.

Translation of alleles ^*^01 of F, ORF, and in-frame P are aligned according to the IMGT unique numbering in IMGT Protein display allowing the visualization of the FR-IMGT and CDR-IMGT [IMGT Repertoire (IG and TR) > 2. Proteins and alleles > Protein displays > IGHV > Atlantic salmon (*S. salar*)]^1^ and the comparison of the CDR-IMGT lengths per subgroup (3) {IMGT Repertoire (IG and TR) > 3. 2D and 3D structures > FR-IMGT and CDR-IMGT lengths (V-REGION and V-DOMAIN) > [CDR1-IMGT.CDR2-IMGT.] length per subgroup > IGHV > Atlantic salmon (*S. salar*)}^1^ (3).

### Rainbow Trout IGH Variable Genes

**Figure 3:**
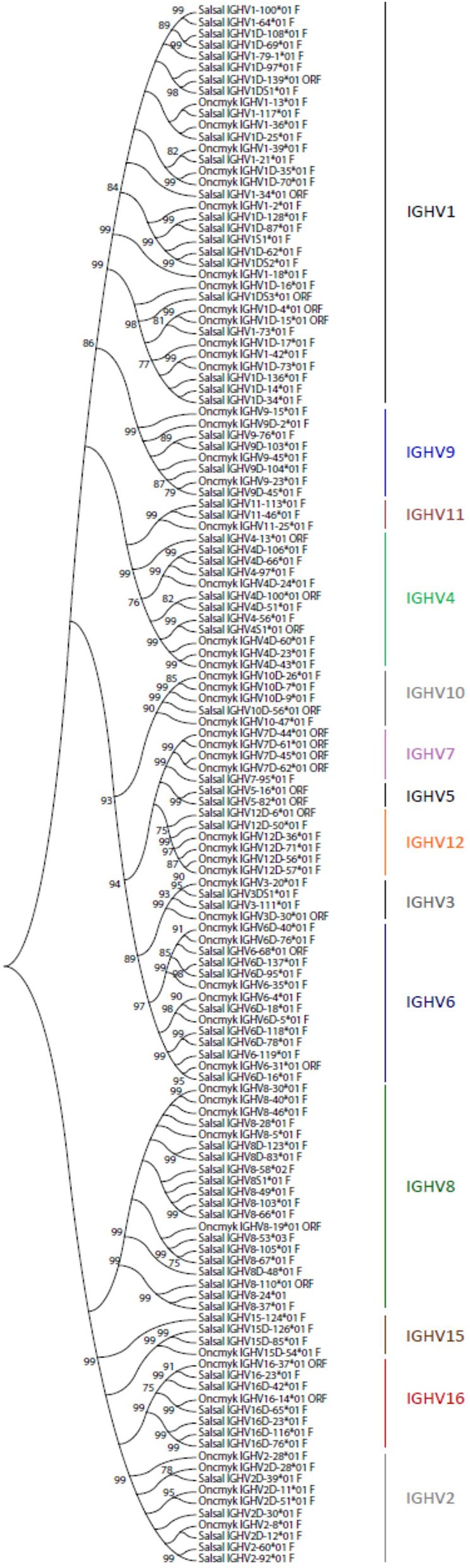
Phylogenetic analysis of Atlantic salmon and rainbow trout IGHV gene sequences. A phylogenetic analysis of the V-REGION of Atlantic salmon (Salsal) and rainbow trout (Oncmyk) IGHV genes (F + ORF) was performed using the UPGMA method and a bootstrap based on 1,000 replicates. A total of 136 genes, 81 from Salsal (69 F + 12 ORF) and 55 from Oncmyk (44 F + 11 ORF) from the IMGT/V-QUEST reference directory sets (release 201931-4, 1st August 2019) (Table 5), were compared. Only one allele per gene was included in the analysis (allele ^*^01 for all but two IGHV8-58^*^02 F and IGHV8-53^*^03 F). Nodes with a bootstrap support higher than 75% are indicated.

A total of 129 IGHV genes were identified in the rainbow trout genome, of which 57 can be considered fully functional or with an ORF without stop codon. A number of other sequences were identified as IGHV fragments in the assembly and were not included in the annotation. On chromosome 13 (locus A), 44 IGHV genes were found upstream of the functional IGHT2 gene, as well as 5 IGHV genes between the D-J-IGHT2 cluster and the D-J-IGHM-IGHD cluster. Eighty IGHV genes were found on chromosome 12 (locus B): 70 IGHV were located upstream of the functional IGHT1D gene and 10 IGHV were found between the D-J-IGHT1D cluster and the D-J-IGHMD-IGHDD cluster. The 129 rainbow trout IGHV genes could be classified into the same 16 subgroups defined for the Atlantic salmon IGHV genes, containing from only 1 pseudogene (i.e., IGHV5, IGHV13, and IGHV14 subgroups) to 35 genes, i.e., IGHV1 subgroup, which includes 12 F, 2 ORF, and 21 P IGHV genes. Figure 3 shows a phylogenetic tree based on nucleotide sequences of IGHV genes (F and ORF) present in Atlantic salmon and rainbow trout IGH loci. While some IGHV subgroups are not represented in both species, as far as we know, this tree illustrates how rainbow trout IGHV genes nicely cluster with their Atlantic salmon counterparts.

### Expressed Repertoire Analysis

IMGT/V-QUEST and its high-throughput version, IMGT/HighV-QUEST, can perform analysis of nucleotide sequences of the IG and TR variable domains (21, 22, 26, 27). These tools run against the IMGT/V-QUEST reference directory database that includes several sets (per group and per species) and are built based on the IMGT standards (3) (annotation in IMGT/LIGM-DB, Gene tables, Alignments of alleles, Protein display, entry in IMGT/GENE-DB). The IMGT/V-QUEST sets comprise IMGT reference sequences from all functional (F) and ORF genes and alleles (in Advanced parameters, Selection of IMGT reference directory set “F + ORF”). The sets also include IMGT reference sequences from pseudogenes (P) and alleles with an in-frame V-REGION for versatile genomic analysis (proposed by default, in Advanced parameters IMGT reference directory set “F + ORF + in-frame P”).

Altogether, IMGT/V-QUEST reference directory for Atlantic salmon IGHV contains 150 alleles that include 76 F, 15 ORF, and 59 P in-frame (release: 201931-4, 1st August 2019) (Table 5). The 76 F comprise, in addition to the 67 F alleles ^*^01 (28 from locus A and 39 from locus B), 8 alleles ^*^02 and 1 allele ^*^03. The 15 ORF comprise, in addition to the 12 ORF alleles ^*^01 (7 from locus A and 5 from locus B), 3 alleles ^*^02. The 59 in-frame P comprise, in addition to the 54 P alleles ^*^01 (26 from locus A and 28 from locus B), 5 alleles ^*^02. Alleles of closely related duplicated genes are managed in the same Alignments of alleles, as shown, for example, for IGHV1-64^*^01 F and IGHV1-100^*^01 F, which have identical V-REGION nucleotide sequences [IMGT Repertoire (IG and TR) 2. Proteins and alleles > Alignments of alleles > IGHV > Atlantic salmon (*S. salar*)]^1^.

**Table 5:** Atlantic salmon (*S. salar*) and rainbow trout (*O. mykiss*) IGHV alleles included in the IMGT/V-QUEST reference directory sets (release 201931-4, 1st August 2019).

**IGHV subgroup**	**Atlantic salmon**								**Rainbow trout**							
	**Nb of genes**	**Nb of alleles**	**IGH locus A**			**IGH locus B**			**Nb of genes**	**Nb of alleles**	**IGH locus A**			**IGH locus B**		
			F^*^	**ORF^**+**^**	P^*^	F^*^	ORF^*^	P^*^			F^*^	ORF^*^	P^*^	F^*^	ORF^*^	P^*^
IGHV1	33	38	7	1(2)	5(6)	12(14)	2	6(7)	17	17	6	0	2	5	2	2
IGHV2	5	5	2	0	0	3	0	0	8	8	2	0	1	3	0	2
IGHV3	3	4	1(2)	0	0	1	0	1	2	2	1	0	0	0	1	0
IGHV4	14	14	2	2	4	3	1	2	9	9	0	0	1	4	0	4
IGHV5	2	3	0	2(3)	0	0	0	0	0	0	0	0	0	0	0	0
IGHV6	14	16	1(2)	1	3	6	0	3(4)	8	8	2	1	0	3	0	2
IGHV7	3	3	1	0	1	0	0	1	4	4	0	0	0	0	4	0
IGHV8	16	20	11(14)	1(2)	1	3	0	0	6	6	4	1	1	0	0	0
IGHV9	8	8	1	0	1	3	0	3	5	5	3	0	1	1	0	0
IGHV10	9	9	0	0	5	0	1	3	4	4	1	0	0	3	0	0
IGHV11	3	4	2	0	1(2)	0	0	0	1	1	1	0	0	0	0	0
IGHV12	2	2	0	0	0	1	1	0	4	4	0	0	0	4	0	0
IGHV13	0	0	0	0	0	0	0	0	0	0	0	0	0	0	0	0
IGHV14	1	1	0	0	1	0	0	0	1	1	0	0	1	0	0	0
IGHV15	6	7	1	0	1	2	0	2(3)	3	3	0	0	0	1	0	2
IGHV16	16	16	1	0	3	5	0	7	5	5	0	2	2	0	0	1
Total	135	150	30(35)	7(10)	26(28)	39(41)	5	28(31)	77	77	20	4	9	24	7	13
Locus A + Locus B	135	150	69(76)F + 12(15)ORF + 54(59)P						77	77	44 F + 11 ORF + 22 P					

* *F, functional; ORF, open reading frame; P, pseudogene. Number of genes included in the IMGT reference directory, per subgroup and per functionality and total, are shown with, if relevant (more than one allele per gene), the corresponding number of alleles within brackets. The functionality is according to IMGT functionality (IMGT Scientific chart > IMGT functionality)^1^ (3)*.

IMGT/V-QUEST reference directory for rainbow trout IGHV contains sequences of 77 alleles that include 44 F, 11 ORF, and 22 P in-frame (/download/V-QUEST/IMGT_V-QUEST_reference_directory/Oncorhynchus_mykiss/IG/IGHV. fasta)^1^. All the alleles are ^*^01 (release: 201931-4, 1st August 2019) (Table 5). All IGHV genes and alleles (including the P out-of-frame) are reported in IMGT Gene tables [IMGT Repertoire (IG and TR) > 1. Locus and genes > Gene tables > IGHV > Rainbow trout (*O. mykiss*)]^1^.

We then investigated the functionality and expression level of IGHV genes from the two species using the standardized nomenclature based on genomic annotation. To do so, adaptive immune receptor repertoire datasets generated by high-throughput sequencing (AIRRseq) were submitted to IMGT/HighV-QUEST analysis.

### Atlantic Salmon

**Figure 4:**
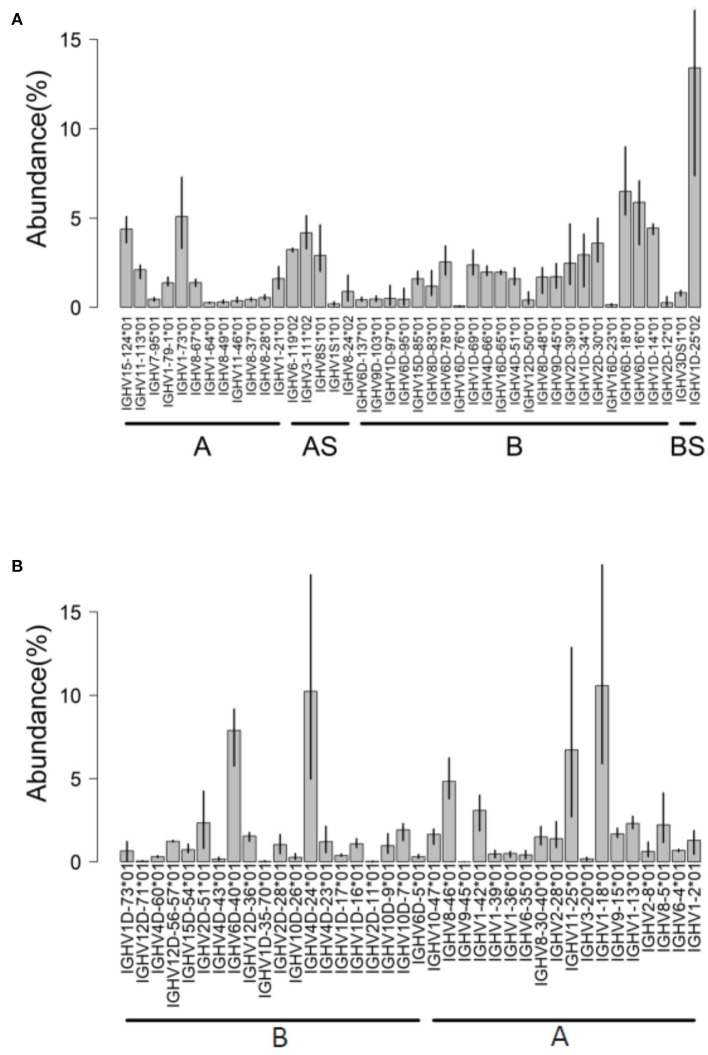
IGHV usage determined by IMGT/HighV-QUEST tool. Analysis of AIRRseq datasets obtained previously from head kidney of three healthy Atlantic salmons **(A)** and from spleen of three rainbow trouts that were intraperitoneally immunized with killed *Yersinia ruckeri* **(B)**. Libraries were generated by 5′RACE using specific primers for IGHM constant region. IGHV usage is expressed as the percentage of total productive rearrangements.

AIRRseq data from head kidney of Atlantic salmon were generated based on 5′RACE and specific primers for IGHM constant region [data from reference (32)]. Using the Atlantic salmon reference dataset updated in 2019, a total of 50 IGHV genes (42 functional “F,” 4 “ORF” and 4 pseudogenes “P”) were expressed in the dataset (Figure 4A). More than 80% of submitted sequences presented IGHV F genes. Interestingly, the majority of expressed V genes were from locus B (chromosome 3). This difference was reflected in the abundance of rearrangements (~66% from locus B) and in the diversity of IGHV genes expressed: 25 IGHV from locus B vs. 17 IGHV from locus A (Figure 4A). On average, IGHV1D-25^*^01, IGHV6D-18^*^01, IGHV6D-16^*^01, and IGHV1-73^*^01 were the most abundant IGHV functional genes, accounting for 30% of the expressed repertoire.

### Rainbow Trout

In this species, we analyzed AIRRseq datasets from fish intraperitoneally immunized with a killed bacterial pathogen, *Yersinia ruckeri* [data from reference (33)]. 5′RACE PCR products were produced from spleen of immunized fish, using specific primers for IGHM constant region and with unique molecular identifiers (UIDs) for better data normalization (33). Only in-frame productive rearrangements (CDR3-IMGT without stop codons) were analyzed. Trout used in this study belonged to the isogenic line derived from Swanson strain that was selected for the rainbow trout genome project. Hence, these AIRRseq data express IGH genes from the very same repertoire, which was annotated in the current IMGT reference directories. These data therefore provided a quantitative assessment of the expression of IGHV genes in the spleen of three genetically similar individuals responding to a pathogen.

In this dataset, IMGT/High V-QUEST unambiguously identified the IGHV gene in 94% of submitted sequences, 90% of them with at least 99% of sequence identity (52% with 100% of identity). A total of 55 IGHV genes (35 functional “F,” 9 “ORF,” and 7 pseudogenes “P”) were expressed. Interestingly, these rearrangements are from both IGH loci (A and B) in relatively similar proportions.

In each trout sample, about 17% of sequences corresponded to IGHV ORF genes and 1.7–4.7% corresponded to IGHV pseudogenes (most of them correspond to IGHV1D-12^*^01 P or IGHV1-21^*^01 P) involved in-frame junction rearrangements. This feature could be detected because we selected the IMGT/HighV-QUEST directory sets “F + ORF + in-frame P,” which also include pseudogenes with in-frame stop codon in V region or defect in the leader or recombination signal (RS) sequences (3). Although IGH transcripts with stop codon are generally rare in mammals, they are typically much more frequent in fish, perhaps because nonsense-mediated mRNA decay (NMD) may work differently (28, 32, 34, 35).

Hence, about 80% of submitted rainbow trout sequences presented functional IGHV genes (Figure 4B). IGHV4D-24^*^01 F, IGHV6D-40^*^01 F, IGHV1-18^*^01 F, and IGHV11-25^*^01 F were the most expressed on average, with a limited interindividual variation as expected from the genetic constitution of the fish analyzed. In this dataset, for about 6% of submitted sequences, IMGT/HighV-QUEST provided two results assigned to distinct duplicated germline IGHV with alleles having identical or close sequences (for example, IGHV12D56^*^01/IGHV12D57^*^01, or IGHV8-30^*^01/IGHV8-40^*^01) owing to the gene duplication in salmonids.

Although the datasets analyzed here for salmon and trout were not selected for direct comparison, it suggests that these two species (at least, the fish strains analyzed here) do not use the two loci in the same way (see above). A rigorous and comprehensive comparison of expressed repertoires between rainbow trout and Atlantic salmon will require a systematic comparison of AIRRseq data from multiple strains.

### Genetic Variability of IG Genes in Salmonids

Making available a full annotation and versatile nomenclature also offers the possibility to better integrate new data about variability of IG (or TR) genes. This issue is of particular interest in Salmonids for two main reasons: (1) variations of IG gene sequences may affect the repertoire of specificities targeted by Abs, in turn impacting the quality and efficiency of responses against pathogens, and (2) salmonid IG loci are particularly complex with high numbers of functional genes and pseudogenes located in two regions; therefore, they constitute interesting models to understand mechanisms of short-term evolution of such loci and the potential importance of homogenization vs. diversification of IG sequences.

To get preliminary data about IGHV variation in a salmonid species, we took advantage of the full genome sequencing of 19 isogenic lines of rainbow trout. These lines were produced using a mitogynogenesis-based strategy by Quillet et al. (36, 37). They represent 19 haplotypes randomly picked from the so-called INRA-SY “synthetic” population. This population was created about 35 years ago by a planned random mating (i.e., panmictic) mixture of French, Danish, and American domestic populations, and has been maintained since without any voluntary selection. The 19 isogenic lines analyzed here do not appear to be closely related to the Swanson trout generated at Washington State University using androgenesis, which has been sequenced and constitutes the reference genome (38, 39).

The numbers of indel and SNP detected within IGHV genes and pseudogenes are indicated in Table 6. Genetic variation between isogenic lines overall appears to be relatively modest at this level. It seems to be more frequent in the locus located in chromosome 13 (67 SNP and 1 indel for 29 functional genes, 41 SNP and 3 indel for 20 pseudogenes) compared to chromosome 12 (23 SNP and no indel for 29 functional genes, and 53 SNP and 10 indel for 51 pseudogenes). The proportion of silent vs. non-silent mutations was not significantly different between the two regions (40NS/67 SNP for chromosome 13 and 13NS/23 SNP for chromosome 12), suggesting that these genes did not evolve under strong positive selection. Indel and SNP were not significantly more frequent in pseudogenes. Variants were filtered to eliminate all assembly artifacts, but these data will have to be fully validated by resequencing, and the impact of variation on the gene status evaluated. We have indications that several new genes are present in productive and expressed rearrangements. This might be due to the absence of such genes in the genome of the Swanson strain or to gaps in the current reference genome assembly. In this context, it is of interest to evaluate the variability of IGHV gene numbers between the different haplotypes. Future assemblies will allow a more accurate description of the IGH diversity and variability. Incompleteness of the annotated repertoire may constitute a problem for repertoire analysis (for example, when a missing gene is used by a clonotype clonally selected in a response). Hence, sequences of genes that are not localized in the current assembly may be added to the IMGT Reference directories sets, providing that sufficient evidence is available to demonstrate their existence and expression. These sequences will be given a provisional name (with S) until their location and presence in the germline genomic sequence are validated. If new genes would appear, which do not belong to any of the IGHV subgroups identified and described in this work, a new subgroup may have to be defined. This is not impossible, but seems to be unlikely since we believe that the large set of IGHV sequences analyzed from Atlantic salmon and rainbow trout probably contains at least one representative of all subgroups. Such additions will be validated by the IG, TR, and MH Nomenclature Sub-Committee (IMGT-NC) (6, 7) of the IUIS Nomenclature Committee^2,3^, following a procedure analogous to the one used for example for inferred alleles in human.

**Table 6:** Number of SNP and variants in IGHV genes and pseudogenes across 19 isogenic rainbow trout lines.

	**Functional genes**					**Pseudogenes**				
**Chrom**	**Start**	**Stop**	**Name**	**SNPs number (NS)**	**Indel number**	**Start**	**Stop**	**Name**	**SNPs number**	**Indel number**
**(A)**										
Chr12	81 322 385	81 322 680	IGHV6D-76	2(1)	0	81 312 817	81 313 128	IGHV16D-79	3	0
Chr12	81 335 727	81 336 024	IGHV1D-73	2(2)	0	81 318 367	81 318 661	IGHV15D-78	5	1
Chr12	81 339 680	81 339 363	IGHV12D-71	1(0)	0	81 320 821	81 321 111	IGHV1D-77	1	0
Chr12	81 365 089	81 365 411	IGHV15D-69	0	0	81 332 711	81 333 058	IGHV3D-75	2	2
Chr12	81 365 950	81 366 255	IGHV2D-68	0	0	81 333 678	81 333 986	IGHV1D-74	1	0
Chr12	81 395 848	81 396 168	IGHV7D-62	0	0	81 336 977	81 337 276	IGHV4D-72	0	0
Chr12	81 397 765	81 398 073	IGHV4D-60	0	0	81 383 887	81 384 159	IGHV1D-65	0	0
Chr12	81 422 492	81 422 803	IGHV15D-54	0	0	81 384 684	81 384 968	IGHV6D-64	0	0
Chr12	81 436 861	81 437 166	IGHV2D-50	0	0	81 388 213	81 388 533	IGHV1D-63	1	1
Chr12	81 438 847	81 439 169	IGHV15D-49	0	0	81 396 766	81 397 086	IGHV7D-61	0	0
Chr12	81 464 500	81 464 820	IGHV7D-45	0	0	81 398 323	81 398 601	IGHV1D-59	0	0
Chr12	81 465 762	81 466 082	IGHV7D-44	0	0	81 399 513	81 399 812	IGHV4D-58	0	0
Chr12	81 466 761	81 467 069	IGHV4D-43	0	0	81 401 688	81 402 032	IGHV12D-57	0	0
Chr12	81 493 829	81 494 124	IGHV6D-40	0	0	81 402 044	81 401 727	IGHV12D-56	0	0
Chr12	81 529 173	81 529 478	IGHV1D-35	0	0	81 421 382	81 421 689	IGHV16D-55	11	0
Chr12	81 561 752	81 562 063	IGHV3D-30	0	0	81 434 530	81 434 810	IGHV2D-52	0	0
Chr12	81 568 866	81 569 171	IGHV2D-28	0	0	81 435 683	81 435 988	IGHV2D-51	0	0
Chr12	81 595 836	81 596 138	IGHV10D-26	2(0)	0	81 447 972	81 448 244	IGHV1D-48	0	0
Chr12	81 605 108	81 605 419	IGHV4D-24	1(0)	0	81 448 773	81 449 068	IGHV6D-47	0	0
Chr12	81 618 381	81 618 689	IGHV4D-23	2(2)	0	81 453 306	81 453 632	IGHV1D-46	0	0
Chr12	81 649 810	81 650 108	IGHV1D-17	5(3)	0	81 467 316	81 467 588	IGHV1D-42	0	0
Chr12	81 653 372	81 653 678	IGHV1D-16	0	0	81 481 949	81 482 241	IGHV16D-41	0	0
Chr12	81 661 001	81 661 301	IGHV1D-15	0	0	81 511 235	81 511 555	IGHV1D-39	2	0
Chr12	81 675 466	81 675 769	IGHV1D-12	2(2)	0	81 514 353	81 514 683	IGHV1D-38	0	0
Chr12	81 696 523	81 696 828	IGHV2D-11	1(0)	0	81 516 973	81 517 272	IGHV4D-37	0	0
Chr12	81 700 731	81 701 033	IGHV10D-9	0	0	81 519 248	81 519 565	IGHV12D-36	1	1
Chr12	81 705 764	81 706 066	IGHV10D-7	2(1)	0	81 548 116	81 548 425	IGHV16D-34	0	0
Chr12	81 717 198	81 717 494	IGHV6D-5	3(2)	0	81 550 863	81 551 152	IGHV16D-33	0	0
Chr12	81 737 673	81 737 973	IGHV1D-4	0	0	81 558 855	81 559 137	IGHV15D-32	0	0
						81 560 796	81 561 107	IGHV12D-31	0	0
						81 567 432	81 567 712	IGHV2D-29	0	0
						81 594 924	81 595 202	IGHV6D-27	1	0
						81 598 873	81 599 174	IGHV1D-25	0	0
						81 618 939	81 619 217	IGHV1D-22	11	2
						81 628 800	81 629 081	IGHV1D-21	2	0
						81 629 835	81 630 118	IGHV8D-20	0	0
						81 630 920	81 631 246	IGHV6D-19	0	0
						81 637 541	81 637 838	IGHV6D-18	0	0
						81 674 597	81 674 901	IGHV4D-13	0	0
						81 699 366	81 699 678	IGHV6D-10	0	0
						81 704 399	81 704 711	IGHV6D-8	0	0
						81 713 243	81 713 531	IGHV6D-6	3	1
						81 745 526	81 745 784	IGHV1D-3	2	0
						81 746 308	81 746 602	IGHV9D-2	1	0
						81 750 741	81 751 044	IGHV16D-1	6	2
						81 671 030	81 671 329	IGHV1D-14	0	0
						81 367 086	81 367 437	IGHV1D-67	0	0
						81 367 599	81 367 922	IGHV15D-66	0	0
						81 430 263	81 430 605	IGHV15D-53	0	0
						81 359 137	81 359 442	IGHV1D-70	0	0
						81 676 641	81 676 923	IGHV5D-11	0	0
Total				23(13)	0				53	10
**(B)**										
Chr13	48 030 797	48 031 104	IGH IGHV10-47	2(1)	0	48 138 071	48 138 427	IGHV8-29	0	0
Chr13	48 034 515	48 034 814	IGHV8-46	1(0)	0	48 027 352	48 027 666	IGHV15-48	7	0
Chr13	48 054 181	48 054 484	IGHV1-42	0	0	48 046 874	48 047 207	IGHV9-45	1	0
Chr13	48 073 234	48 073 536	IGHV8-40	0	0	48 048 080	48 048 362	IGHV4-44	0	0
Chr13	48 077 115	48 077 414	IGHV1-39	1(1)	0	48 051 342	48 051 671	IGHV1-43	2	0
Chr13	48 082 080	48 082 391	IGHV16-37	3(3)	0	48 068 027	48 068 332	IGHV1-41	0	0
Chr13	48 093 298	48 093 597	IGHV1-36	0	0	48 079 966	48 080 277	IGHV16-38	1	0
Chr13	48 104 897	48 105 217	IGHV6-35	0	0	48 108 554	48 108 889	IGHV9-34	0	0
Chr13	48 109 683	48 109 994	IGHV14-33	0	0	48 146 928	48 147 231	IGHV2-27	3	0
Chr13	48 122 499	48 122 783	IGHV6-32	0	0	48 147 810	48 148 106	IGHV6-26	3	0
Chr13	48 127 168	48 127 466	IGHV6-31	0	0	48 157 816	48 158 168	IGHV9-24	5	2
Chr13	48 135 329	48 135 631	IGHV8-30	0	0	48 211 869	48 212 178	IGHV7-17	3	0
Chr13	48 145 742	48 146 047	IGHV2-28	7(3)	0	48 244 719	48 245 034	IGHV10-12	3	0
Chr13	48 148 981	48 149 284	IGHV11-25	0	0	48 245 867	48 246 172	IGHV8-11	2	0
Chr13	48 164 983	48 165 317	IGHV9-23	4(3)	0	48 254 879	48 254 570	IGHV16-9	0	0
Chr13	48 166 898	48 167 189	IGHV4-22	9(7)	0	48 279 548	48 279 841	IGHV1-7	0	0
Chr13	48 168 162	48 168 465	IGHV1-21	5(3)	0	48 280 387	48 280 681	IGHV4-6	4	1
Chr13	48 174 816	48 175 127	IGHV3-20	4(2)	0	48 316 059	48 315 761	IGHV6-3	3	0
Chr13	48 191 970	48 192 272	IGHV8-19	6(5)	0	48 339 577	48 339 885	IGHV4-1	0	0
Chr13	48 201 668	48 201 973	IGHV1-18	2(0)	0	48 076 216	48 076 475	IGHV13-39	4	0
Chr13	48 222 441	48 222 126	IGHV9-16	1(1)	1					
Chr13	48 223 844	48 223 544	IGHV9-15	4(2)	0					
Chr13	48 237 588	48 237 899	IGHV16-14	6(6)	0					
Chr13	48 243 688	48 243 987	IGHV1-13	3(0)	0					
Chr13	48 250 487	48 250 789	IGHV1-10	0	0					
Chr13	48 257 525	48 257 828	IGHV2-8	2(1)	0					
Chr13	48 307 972	48 307 670	IGHV8-5	7(2)	0					
Chr13	48 312 160	48 311 865	IGHV6-4	0	0					
Chr13	48 327 417	48 327 761	IGHV1-2	0	0					
Total				67(40)	1				41	3

## Conclusion

Genome assembly is available for both Atlantic salmon and rainbow trout, representing the two main genera of Salmonids (*Salmo* and *Oncorhynchus*). More genomic (and transcriptome) data are coming from a number of genomic backgrounds, which will provide a rich source of knowledge about variations of potential antibody repertoires in these species. We therefore revisited the description and annotation of the two IGH loci present in these two species, currently from cDNA and BAC clone sequences, based on the IMGT biocuration and nomenclature for Salmonid IGH genes that will facilitate the analysis of AIRRseq data.

The IG or antibody repertoire sequencing has started to develop both in rainbow trout and in Atlantic salmon, reflecting a growing interest for an accurate and comprehensive description of the response against common pathogens and vaccines. As full genome assemblies are now available for several salmonid species (Atlantic salmon, rainbow trout, coho salmon, and chinook salmon), comparative analysis of the IGH locus structure in these closely related tetraploidized species is of great interest. It also appears very important to investigate the level of variation between germline repertoires of IG genes across commercial and wild salmonid stocks. This variation may have significant implications for practical issues in aquaculture and conservation; it will also be of significant interest for the basic comparative immunology community, in particular to address accurately the mechanisms of gene conversion, somatic hypermutation, and memory in these species and during vertebrate evolution.

## Data Availability Statement

The datasets generated for this study can be found in the www.imgt.org – accession numbers can be found within the manuscript. Any other data supporting the conclusions of this manuscript will be made available by the authors, without undue reservation, to any qualified researcher.

## Author Contributions

SMa, AK, M-PL, and PB conceived the project and wrote the manuscript. SMa, AK, IS, and PB designed experiments. SMa, AK, SH-S, SA, SMo, DL, RC, IS, OS, JH, BK, M-PL, and PB performed data analysis. SMa, AK, DL, and IS provided resources. All authors contributed to manuscript revision, and read and approved the submitted version.

### Conflict of Interest

The authors declare that the research was conducted in the absence of any commercial or financial relationships that could be construed as a potential conflict of interest.

## Footnotes

^1^http://www.imgt.org

^2^http://www.imgt.org/IMGTindex/IMGT-NC.php

^3^http://www.imgt.org/IMGTindex/IUIS-NC.php

## Supplementary Material

Figure S1 Alignment of the D-GENE-UNIT sequences of the IGHD (diversity) genes located upstream of the IGHM (locus A) and IGHMD (locus B) genes of *Salmo salar* (Salsal) and *Oncorhynchus mykiss* (Oncmyk) **(A)** and located upstream of IGHT genes **(B)**. Genes of the locus B genes are identified by the letter D which follows the gene number. Labels are according to the D-GENE prototype (IMGT Scientific chart > 1. Sequence and 3D structure identification and description > IMGT prototypes table > D-GENE)^1^.

Figure S2 Alignment of the J-REGION amino acid sequences of the IGHJ (joining) genes located upstream of the IGHM or IGHT (locus A) and IGHMD or IGHTD (locus B) genes of Salmo salar (Salsal) and *Oncorhynchus mykiss* (Oncmyk). Genes of the locus B are identified by the letter D which follows the gene number. Labels are according to the J-GENE prototype (IMGT Scientific chart > 1. Sequence and 3D structure identification and description > IMGT prototypes table > J-GENE)^1^. The highly conserved FDYWGKGTXVT motif is pink highlighted and those residues that deviated from it are in red.

## References

[1] Lefranc M-P Lefranc G The Immunoglobulin FactsBook. London, UK: Academic Press (2001).

[2] Lefranc M-P Lefranc G The T Cell Receptor FactsBook. London, UK: Academic Press (2001).

[3] Lefranc M-P. Immunoglobulin and T cell receptor genes: IMGT® and the birth and rise of immunoinformatics. Front Immunol. (2014) 5:22. 10.3389/fimmu.2014.00022 24600447 PMC3913909

[4] Lefranc M-P Nomenclature of the human immunoglobulin genes. Curr Protoc Immuno. (2001) Appendix1: Appendix 1P.1-A1P37. 10.1002/0471142735.ima01ps40 18432650

[5] Lefranc M-P Nomenclature of the human T cell receptor genes. Curr Protoc Immunol. (2001) Appendix 1:Appendix 1O. 1-A.1O.23. 10.1002/0471142735.ima01os40 18432649

[6] Lefranc M-P. WHO-IUIS Nomenclature Subcommittee for immunoglobulins and T cell receptors report. Immunogenetics. (2007) 59:899–902. 10.1007/s00251-007-0260-4 18046549

[7] Lefranc M-P. WHO-IUIS nomenclature subcommittee for immunoglobulins and T cell receptors report August 2007, 13th International Congress of Immunology, Rio de Janeiro, Brazil. Dev Comp Immunol. (2008) 32:461–3. 10.1016/j.dci.2007.09.008 18036660

[8] Matsunaga T Chen T Törmänen V. Characterization of a complete immunoglobulin heavy-chain variable region germ-line gene of rainbow trout. Proc Natl Acad Sci USA. (1990) 87:7767–71. 10.1073/pnas.87.19.7767 2120708 PMC54829

[9] Andersson E Törmänen V Matsunaga T. Evolution of a VH gene family in low vertebrates. Int Immunol. (1991) 3:527–33. 10.1093/intimm/3.6.527 1716145

[10] Lee MA Bengtén E Daggfeldt A Rytting AS Pilström L. Characterisation of rainbow trout cDNAs encoding a secreted and membrane-bound Ig heavy chain and the genomic intron upstream of the first constant exon. Mol Immunol. (1993) 30:641–8. 10.1016/0161-5890(93)90075-M 8487781

[11] Andersson E Matsunaga T. Complete cDNA sequence of a rainbow trout IgM gene and evolution of vertebrate IgM constant domains. Immunogenetics. (1993) 38:243–50. 10.1007/BF00188800 8319974

[12] Roman T Charlemagne J. The immunoglobulin repertoire of the rainbow trout (Oncorhynchus mykiss): definition of nine Igh-V families. Immunogenetics. (1994) 40:210–6. 10.1007/BF00167081 8039829

[13] Roman T Andersson E Bengtén E Hansen J Kaattari S Pilström L. Unified nomenclature of Ig VH genes in rainbow trout (*Oncorhynchus mykiss*): definition of eleven VH families. Immunogenetics. (1996) 43:325–6. 10.1007/s002510050072 9110939

[14] Brown GD Kaattari IM Kaattari SL. Two new Ig VH gene families in *Oncorhynchus mykiss*. Immunogenetics. (2006) 58:933–6. 10.1007/s00251-006-0149-7 17039360

[15] Solem ST Hordvik I Killie JA Warr GW Jørgensen TO. Diversity of the immunoglobulin heavy chain in the Atlantic salmon (Salmo salar L.) is contributed by genes from two parallel IgH isoloci. Dev Comp Immunol. (2001) 25:403–17. 10.1016/S0145-305X(01)00008-8 11356220

[16] Yasuike M de Boer J von Schalburg KR Cooper GA McKinnel L Messmer A. Evolution of duplicated IgH loci in Atlantic salmon, Salmo salar. BMC Genom. (2010) 11:486. 10.1186/1471-2164-11-486 20813058 PMC2996982

[17] Lefranc M-P Giudicelli V Duroux P Jabado-Michaloud J Folch G Aouinti S. IMGT®, the international ImMunoGeneTics information system® 25 years on. Nucleic Acids Res. (2015) 43:D413–22. 10.1093/nar/gku1056 25378316 PMC4383898

[18] Li S Lefranc M-P Miles JJ Alamyar E Giudicelli V Duroux P. IMGT/HighV QUEST paradigm for T cell receptor IMGT clonotype diversity and next generation repertoire immunoprofiling. Nat Commun. (2013) 4:2333. 10.1038/ncomms3333 23995877 PMC3778833

[19] Aouinti S Malouche D Giudicelli V Kossida S Lefranc M-P. IMGT/HighV-QUEST statistical significance of IMGT clonotype (AA) diversity per gene for standardized comparisons of next generation sequencing immunoprofiles of immunoglobulins and T cell receptors. PLoS ONE. (2015) 10:e0142353. 10.1371/journal.pone.0142353 26540440 PMC4634997

[20] Aouinti S Giudicelli V Duroux P Malouche D Kossida S Lefranc M-P. IMGT/statclonotype for pairwise evaluation and visualization of NGS IG and TR IMGT clonotype (AA) diversity or expression from IMGT/HighV-QUEST. Front Immunol. (2016) 7:339. 10.3389/fimmu.2016.00339 27667992 PMC5016520

[21] Brochet X Lefranc M-P Giudicelli V. IMGT/V-QUEST: the highly customized and integrated system for IG and TR standardized V-J and V-D-J sequence analysis. Nucleic Acids Res. (2008) 36:W503–8. 10.1093/nar/gkn316 18503082 PMC2447746

[22] Monod MY Giudicelli V Chaume D Lefranc M-P IMGT/JunctionAnalysis: the first tool for the analysis of the immunoglobulin and T cell receptor complex V-J and V-D-J JUNCTIONs. Bioinformatics. (2004) 20 (Suppl_1):i379–85. 10.1093/bioinformatics/bth945 15262823

[23] Ehrenmann F Lefranc M-P. IMGT/DomainGapAlign: the IMGT® tool for the analysis of IG, TR, MH, IgSF, and MhSF domain amino acid polymorphism. Methods Mol Biol. (2012) 882:605–33. 10.1007/978-1-61779-842-9_33 22665257

[24] Lefranc M-P Pommié C Ruiz M Giudicelli V Foulquier E Truong L. IMGT unique numbering for immunoglobulin and T cell receptor variable domains and Ig superfamily V-like domains. Dev Comp Immunol. (2003) 27:55–77. 10.1016/S0145-305X(02)00039-3 12477501

[25] Lefranc M-P Pommié C Kaas Q Duprat E Bosc N Guiraudou D. IMGT unique numbering for immunoglobulin and T cell receptor constant domains and Ig superfamily C-like domains. Dev Comp Immunol. (2005) 29:185–203. 10.1016/j.dci.2004.07.003 15572068

[26] Alamyar E Duroux P Lefranc M-P Giudicelli V. IMGT® tools for the nucleotide analysis of immunoglobulin (IG) and T cell receptor (TR) V-(D)-J repertoires, polymorphisms, and IG mutations: IMGT/V-QUEST and IMGT/HighV-QUEST for NGS. Methods Mol Biol. (2012) 882:569–604. 10.1007/978-1-61779-842-9_32 22665256

[27] Alamyar E Giudicelli V Shuo L Duroux P Lefranc M-P. IMGT/HighV-QUEST: the IMGT® web portal for immunoglobulin (IG) or antibody and T cell receptor (TR) analysis from NGS high throughput and deep sequencing. Immunome Res. (2012) 8:26. 22665256

[28] Fillatreau S Six A Magadan S Castro R Sunyer JO Boudinot P. The astonishing diversity of Ig classes and B cell repertoires in teleost fish. Front Immunol. (2013) 4:28. 10.3389/fimmu.2013.00028 23408183 PMC3570791

[29] Hansen JD Landis ED Phillips RB. Discovery of a unique Ig heavy-chain isotype (IgT) in rainbow trout: implications for a distinctive B cell developmental pathway in teleost fish. Proc Natl Acad Sci USA. (2005) 102:6919–24. 10.1073/pnas.0500027102 15863615 PMC1100771

[30] Zhang Y-A Salinas I Li J Parra D Bjork S Xu Z. IgT, a primitive immunoglobulin class specialized in mucosal immunity. Nat Immunol. (2010) 11:827–35. 10.1038/ni.1913 20676094 PMC3459821

[31] Hordvik I. Identification of a novel immunoglobulin delta transcript and comparative analysis of the genes encoding IgD in Atlantic salmon and Atlantic halibut. Mol Immunol. (2002) 39:85–91. 10.1016/S0161-5890(02)00043-3 12213331

[32] Krasnov A Jørgensen SM Afanasyev S. Ig-seq: deep sequencing of the variable region of Atlantic salmon IgM heavy chain transcripts. Mol Immunol. (2017) 88:99–105. 10.1016/j.molimm.2017.06.022 28623734

[33] Magadan S Jouneau L Boudinot P Salinas I. Nasal vaccination drives modifications of nasal and systemic antibody repertoires in rainbow trout. *J Immunol*. (2019) J Immunol. 203:1480–92. 10.4049/jimmunol.1900157 31413108

[34] Castro R Jouneau L Pham H-P Bouchez O Giudicelli V Lefranc M-P. Teleost fish mount complex clonal IgM *and* IgT responses in spleen upon systemic viral infection. PLoS Pathog. (2013) 9:e1003098. 10.1371/journal.ppat.1003098 23326228 PMC3542120

[35] Magadán-Mompó S Sánchez-Espinel C Gambón-Deza F. Immunoglobulin heavy chains in medaka (*Oryzias latipes*). BMC Evol Biol. (2011) 11:165. 10.1186/1471-2148-11-165 21676244 PMC3141427

[36] Quillet E Dorson M Leguillou S Benmansour A Boudinot P. Wide range of susceptibility to rhabdoviruses in homozygous clones of rainbow trout. Fish Shellfish Immunol. (2007) 22:510–9. 10.1016/j.fsi.2006.07.002 17085058

[37] Diter A Quillet E Chourrout D Suppression of first egg mitosis induced by heat shocks in the rainbow trout. J Fish Biol. (1993) 42:777–86. 10.1111/j.1095-8649.1993.tb00383.x

[38] Palti Y Genet C Luo M-C Charlet A Gao G Hu Y. A first generation integrated map of the rainbow trout genome. BMC Genom. (2011) 12:180. 10.1186/1471-2164-12-180 21473775 PMC3079668

[39] Palti Y Gao G Miller MR Vallejo RL Wheeler PA Quillet E. A resource of single-nucleotide polymorphisms for rainbow trout generated by restriction-site associated DNA sequencing of doubled haploids. Mol Ecol Resour. (2014) 14:588–96. 10.1111/1755-0998.12204 24251403

