# Supplementary material for: Standardized IMGT® Nomenclature of Salmonidae IGH Genes, the Paradigm of Atlantic Salmon and Rainbow Trout: From Genomics to Repertoires
Source: Front Immunol. 2019 Nov 12;10:2541. doi: 10.3389/fimmu.2019.02541 (PMC6866254; doi:10.3389/fimmu.2019.02541)
Supplement: Figure S1 — Alignment of the D-GENE-UNIT sequences of the IGHD (diversity) genes located upstream of the IGHM (locus A) and IGHMD (locus B) genes of Salmo salar (Salsal) and Oncorhynchus mykiss (Oncmyk) (A) and located upstream of IGHT genes (B). Genes of the locus B genes are identified by the letter D which follows the gene number. Labels are according to the D-GENE prototype (IMGT Scientific chart > 1. Sequence and 3D structure identification and description > IMGT prototypes table > D-GENE)1. [file Data_Sheet_1.PDF]

A)

	5´D-RS			IGHD	3´D-RS		
	5´D-NONAMER	5´D-SPACER	5´D-HEPTAMER	D-REGION	5´D-HEPTAMER	5´D-SPACER	5´D-NONAMER
**Salsal IGHD1*01**	GGTTTTTGT	ATGGCTGT-GTGT	CATCGTG	ATAACTACGGA-----GG	CACAGTG	TTGCACGCCTTA	ACAAAAACC
**Salsal IGHD1D*01**	AGTTTTTGT	ATGGGTGT-GTGT	CATCGTG	ATAACTACGGG-----GG	CACAGTG	TTACACTCCATA	ACAAAAACC
**Oncmyk IGHD1*01**	GGTTTTTGT	ATGGGTGT-GTGT	CATCGTG	ATAACTACGGG-----GG	CACAGTG	TTACAAACCATA	ACAAAAACC
**Oncmyk IGHD1D*01**	GGTTTTTGT	ATGGGTGT-GTGT	CATCGTG	ATAACTACGGG-----GG	CACAGTG	TTACAAACCATA	ACAAAAACC
**Salsal IGHD2*01**	GGTTTTTGT	ACAGGGTT-AAAC	CACTGTG	CCATATAGCGG-----GT	CACAATG	ATACAGTCCATG	ACAAAAACC
**Salsal IGHD2D*01**	GGTTTTTGT	ACAGGGTT-AAAC	CACTGTG	CCATATAGCGG-----GT	CACAATG	ATACAGTCCATG	ACAAAAACC
**Oncmyk IGHD2*01**	GGTTTTTGT	ACAGGGTT-AAAC	CACTGTG	CCATATAGCGG----GGT	CACAATG	ATACAGTCCATG	ACAAAAACC
**Oncmyk IGHD2D*01**	GGTTTTTGT	ACAGGGTT-AAAC	CACTGTG	TCATATAGCGG-----GT	CACAATG	ATACAGTCCATG	ACAAAAACC
**Salsal IGHD3*01**	GGTTTTTGT	AATGGTGT-GAGT	CACTGTG	-----TATGGG-AATGGC	CACAGTG	ATCAACATCAAT	ACAAAAACC
**Salsal IGHD3D*01**	GGTTTTTGT	AATGGTGT-GAGT	CACTGTG	-----TATGGG-AATGGC	CACAGTG	ATAAACATCAAT	ACAAAAACC
**Oncmyk IGHD3D*01**	GGTTTTTGT	AATGGTGT-GAGT	CACTGTG	-----TACGGG-AATGGC	CACAGTG	ATACACATCAAT	ACTAAAACC
**Oncmyk IGHD3*01**	GGTTTTTGT	AATGGTGT-GATT	CACTGTG	-----TATGGG-AGTGGC	CACAGTG	ATAAACATCAAT	ACTAAAACC
**Salsal IGHD9*01**	GGTTTTTGT	AATGGTGT-GTGT	CATTGTG	-----TATGGG-GGCAGC	CACAGTG	ATACACACCAAT	ACAAAAA--
**Salsal IGHD6D*01**	GGTTTTTGT	AATGGTGT-GTGT	CATTGTG	-----TATGGG-GGCAGC	CACAGTG	ATACCCACCAAT	ACAAAAACC
**Salsal IGHD6*01**	GGTTTTTGT	AATGGTGT-GTGT	CACTGTG	-----TATGGG-GGCAGC	CACAGTG	ATATACACCAAT	ACAAAAACC
**Oncmyk IGHD6*01**	GGTTTTTGT	AATGATGT-GAGT	CACTGTG	-----TATGGG-GGCAGC	CACAGTG	ATACACACCACT	ACGAAAACC
**Oncmyk IGHD6D*01**	GGTTTTTGT	AATGGTGT-GTGC	CACTGTG	-----TATGGG-GGCAGC	CACAGTG	ATACACACCACT	ACGAAAACC
**Salsal IGHD8*01**	AGTTTTTGT	ACTGGCAT-GTAT	CACTGTG	-----TACACT-GGGAGC	CACAATG	ATATACACCAGT	ACAAAAACC
**Oncmyk IGHD5*01**	AGATTTTGT	ACTGGCAT-GTAT	CACTGTG	-----TACACT-GGGAGC	CACAATG	ATATACACCAGT	ACAAAAACC
**Oncmyk IGHD5D*01**	AGATTTTGT	ACTGGCAT-GTAT	CACTGTG	-----TACACT-GGGAGC	CACAATG	ATATACACCAGT	ACAAAAACC
**Salsal IGHD5D*01**	AGTTTTTGT	ACTGGCAT-GTAT	CACTGTG	-----TACACT-GGGGGC	CACAATG	ATATACACCAGT	ACAAAAACC
**Salsal IGHD5*01**	AGTTGTTGT	ACTGGCATGGTAT	CACTGTG	-----TACACTGGGGGGC	CACAATG	ATATACACCAGT	ACAAAAACC
**Salsal IGHD4*01**	GGGTTTTGT	ACGGGGAT-GTAT	CACAGTG	-----CAGAAT-AACGGC	CACAGTG	ATACAGCCCATG	ACAAAAACT
**Salsal IGHD7*01**	GGGTTTTGT	ACAGGGAT-GTAT	AACAGTG	-----CAGAAT-AACGGC	CACAGTG	ATACAGCCCATG	AC-AAAACC
**Salsal IGHD4D*01**	GGTTTTTGT	ACAGGGAT-GTAT	CACAGTG	-----CAGAAT-AACGGC	CACAGTG	ATACAGCCCATG	ACAAAAACT
**Oncmyk IGHD4*01**	GGTTTTTGT	ACAGGGAT-GTAT	CACAGTG	-----CAGAAT-AACGGC	CACAGTG	ATATAGCCCATG	ACAAAAACT
**Oncmyk IGHD4D*01**	GGTTTTTGT	ACAGGGAT-GTAT	CACAGTG	-----CAGAAT-AACGGC	CACAGTG	ATATAGCCCATG	ACAAAAACT
**Consensus**	**GGTTTTTGT**	**A--GG--T-G--T**	**CAC-GTG**	**-----TA---------G-**	**CACA-TG**	**ATA-A--CCA--**	**ACAAAAACC**

B)

	5´D-RS			IGHD	3´D-RS		
	5´D-NONAMER	5´D-SPACER	5´D-HEPTAMER	D-REGION	5´D-HEPTAMER	5´D-SPACER	5´D-NONAMER
**Oncmyk IGHD1T2*01**	GGTTTTTGT	TATGGTGTTTTT	CACAGTG	ACTATACAGTTACAGTTTGGGCTTTTTATTCGAGAGC	CACAGTG	ATATTACATCGT	ACAAAAACC
**Salsal IGHD1T2*01**	GGTTTTTGT	TATGGTGTGTAT	CACTGTG	ACTATACAGTTATATTTGGGGTTCTTT----GAGAGC	CACAGTG	ATATTACGCCAT	ACAAAGACC
**Salsal IGHD1T4*01**	GGTTTTTGT	TATGGTGTGTAT	CACTGTG	ACTATACAGTTATATTTGGGGTTCTTT----GAGAGC	CACAGTG	ATATTACGCCAT	ACAAAGACC
**Oncmyk IGHD1T1D*01**	GGTTTTTGT	TATGGTGTGTAT	CACTGTG	ACTATAT------------------------GGGGGC	CACAGTG	ATATTACACCGT	ACAAAAACC
**Oncmyk IGHD1T1*01**	GGTTTTTGT	TATGGTGTGTAT	CACTGTG	ACTATAT------------------------GGGGGC	CACAGTG	ATATTACGCCGT	ACAAAAACC
**Salsal IGHD1T2D*01**	AGTTTTTGT	TATGGTGTTTAT	CACTGTG	ACTATAT------------------------GGGGGT	CACAGTG	ATATTACGTCGT	ACAAAAACC
**Salsal IGHD1T5*01**	GGTTTTTGT	TATGGTGTGTAT	CACTGTG	ACTTTAT------------------------GGGGGT	CACAGTG	ATATTACGCCGT	ACAAAAACC
**Salsal IGHD1T2*01**	TGTTTTTGT	GCTGGAGTATAT	CACTGTG	A-TATGGGGTT--------------------TGGGGA	CACAGTG	AAATGAGGCTGT	ACAAAAACC
**Salsal IGHD2T4*01**	TGTTTTTGT	GCTGGAGTATAT	CACTGTG	A-TATGGGGTT--------------------TGGGGA	CACAGTG	AAATGAGGCTGT	ACAAAAACC
**Oncmyk IGHD2T1D*01**	TGTTTTTGT	GCTGGGGTATAT	CACTGTG	A-TATGGGGT---------------------GGGGGG	CACAGTG	AAATGAGGCTGT	ACAAAAACC
**Oncmyk IGHD2T2*01**	TGTTTTTGT	GCTGGGGTATAT	CACTGTG	A-TATGGGGT---------------------GGGGTG	CACAGTG	AAATGAGACTGT	ACAAAAACC
**Oncmyk IGHD2T1*01**	TGTTTTTGT	GCTGGGGTATAT	CACTGTG	A-TATGGGCT---------------------GGGGGG	CACTGTG	AAATGAGGCTGT	ACAAAAACC
**Salsal IGHD2T2D*01**	TGTTTTTGT	GCTGGGGTATAT	CACTGTG	A-TATGGGCT---------------------GGGGGG	CACAGTG	AAATGAGGCTGT	ACAGAAACC
**Salsal IGHD5T4*01**	GGTTTTTGT	AATGGTGTGAGT	CTCTGTG	--TATGGG-----------------------AATGGC	TACAGTG	ATCAACATCAAT	ACAAAAACC
**Oncmyk IGHD4T1D*01**	TGTTTTTGT	AATGGTGTGAGT	CACTGTG	--TACGGG-----------------------AATGGC	CACAGTG	ATAAACATCAAT	ACTAAAACC
**Salsal IGHD3T4*01**	GGTTTTTGT	ATGGGTGTGTGT	CATCATG	ATAACTA------------------------CAAGGG	CACAGTG	TTACACGTCATA	ACAAAAACC
**Salsal IGHD3T3D*01**	GGTTTTTGT	ATGGGTGTGTGT	CATCGTG	ATAA---------------------------CTACGG	CACAGTG	TTACACTCCATA	ACAAAAACC
**Salsal IGHD4T4*01**	GGTTTTTGT	ACAGGGTTAAAC	CACTGTG	C-CATATA-----------------------GCAGGT	CACAATG	ATACAGTCCATG	ACAAAAACC
**Salsal IGHD2T3D*01**	GGTTTTTGT	ACAAGGTTAAAC	CACTGTG	C-CATATA-----------------------GCGGGT	CACAATG	ATACAGTCCATG	ACAAAAACC
**Oncmyk IGHD3T1*01**	GGGGTTTGT	ATAGGGTTAAAC	CACTGTG	C-CATATA-----------------------GCAGGT	CACAATG	ATACAGTCCATG	ACAAAAACC
**Oncmyk IGHD3T1D*01**	GGGAGTTGT	ACAGGGTTAAAC	CACTGTG	C-CACATA-----------------------GCGGGT	CACAATG	ATACAGTCCATG	ACAAAAACC
**Oncmyk IGHD5T1D*01**	TGTATTTGT	ACGGGGATGTAT	CACAGTG	CAGTATAA----------------------—-CAGC-	CACAGTG	ATACACTTCCCC	CTCTCTAAC
**Salsal IGHD1T3D*01**	GGTTTTAGT	AATGGTGTGAAT	CACTGTG	--TACTAG-----------------------AGTGGC	CACAGTG	ATAAACATCAAT	ACAAAAACC
**Consensus**	**-GTTTTTGT**	**---GG-GT--AT**	**CACTGTG**	**---AT--------------------------GG-**	**CACAGTG**	**A-A-----C---**	**ACAAAAACC**
