# Supplementary material for: Standardized IMGT® Nomenclature of Salmonidae IGH Genes, the Paradigm of Atlantic Salmon and Rainbow Trout: From Genomics to Repertoires
Source: Front Immunol. 2019 Nov 12;10:2541. doi: 10.3389/fimmu.2019.02541 (PMC6866254; doi:10.3389/fimmu.2019.02541)
Supplement: Figure S2 — Alignment of the J-REGION amino acid sequences of the IGHJ (joining) genes located upstream of the IGHM or IGHT (locus A) and IGHMD or IGHTD (locus B) genes of Salmo salar (Salsal) and Oncorhynchus mykiss (Oncmyk). Genes of the locus B are identified by the letter D which follows the gene number. Labels are according to the J-GENE prototype (IMGT Scientific chart > 1. Sequence and 3D structure identification and description > IMGT prototypes table > J-GENE)1. The highly conserved FDYWGKGTXVT motif is pink highlighted and those residues that deviated from it are in red. [file Data_Sheet_2.PDF]

| | |
|---|---|
| Oncmyk IGHJ1T1*01 F | AFDYWGKGTQVTVST |
| Oncmyk IGHJ2T1*01 F | YSYFDYWGKGTMVTVSS |
| Oncmyk IGHJ1T2*01 F | AFDYWGKGTQVTVSA |
| Oncmyk IGHJ2T2*01 F | YGYFDYWGKGTMITVSS |
| Oncmyk IGHJ1*01 F | YYFDYWGKGTQVTITS |
| Oncmyk IGHJ2*01 F | NYFDYWGKGTMVTVST |
| Oncmyk IGHJ3*01 F | NAFDYWGKGTMVTVSS |
| Oncmyk IGHJ4*01 F | YRFDYWGKGTMVTVSS |
| Oncmyk IGHJ5*01 F | YAAFDYWGQGTIVTVS |
| Oncmyk IGHJ6*01 F | NAFDYWGKGTMVTVSS |
| Oncmyk IGHJ1T1D*01 F | AFDYWGKGTQVTVST |
| Oncmyk IGHJ2T1D*01 F | YSYFDYWGKGTMVTVSS |
| Oncmyk IGHJ1D*01 F | YYFDYWGKGTQVTITS |
| Oncmyk IGHJ2D*01 F | NAFDYWGKGTMVTVSS |
| Oncmyk IGHJ3D*01 F | YYFDYWGKGTQVTITS |
| Oncmyk IGHJ4D*01 F | NYFDYWGKGTMVTVST |
| Oncmyk IGHJ5D*01 F | NAFDYWGKGTMVTVSS |
| Oncmyk IGHJ6D*01 F | YGFDYWGKGTMVTVSS |
| Salsal IGHJ1*01 F | YYFDYWGKGTQVTITS |
| Salsal IGHJ1-1*01 F | YYFDYWGKGTQVTITS |
| Salsal IGHJ1-2*01 F | NYFDYWGKGTMVTVST |
| Salsal IGHJ1-3*01 F | NAFDHWGKGTMVTVSS |
| Salsal IGHJ1-4*01 F | YGFDYWGKGTMVTVSS |
| Salsal IGHJ1-5*01 F | DAAFDYWGQGTIVTVSL |
| Salsal IGHJ2*01 ORF | NYFDYWGKGTMVTVST |
| Salsal IGHJ2-1*01 F | YYFDYWGKGTQVTITS |
| Salsal IGHJ2-2*01 F | NYFDYWGKGTMVTVST |
| Salsal IGHJ2-3*01 F | HAFDYWGKGTMVTVSS |
| Salsal IGHJ2-4*01 F | YGFDYWGKGTMVTVSS |
| Salsal IGHJ2-5*01 F | DAAFDYWGQGTIVTVSL |
| Salsal IGHJ3*01 F | NAFDHWGKGTMVTVSS |
| Salsal IGHJ4*01 F | YGFDYWGKGTMVTVSS |
| Salsal IGHJ5*01 F | YAAFDYWGQGTIVTVSL |
| Salsal IGHJ1D*01 F | YYFDYWGKGTQVTITS |
| Salsal IGHJ1T2*01 F | AFDYWGKGTQVTVST |
| Salsal IGHJ1T2D*01 F | AFDYWGKGTQVTVST |
| Salsal IGHJ1T3*01 F | AFDYWGKGTQVTIST |
| Salsal IGHJ1T4*01 F | AFDYWGKGTQVTVST |
| Salsal IGHJ2D*01 F | NYFDYWGKGTMVTVST |
| Salsal IGHJ2T2*01 F | YGYFDYWGKGTLVTVSS |
| Salsal IGHJ2T2D*01 F | YGYFDYWGKGTMITVSS |
| Salsal IGHJ2T3*01 F | YGYFDYWGKGTMITVSS |
| Salsal IGHJ2T4*01 F | YGYFDYWGKGTLVTVSS |
| Salsal IGHJ2T5*01 F | YSYFDYWGKGTMVTVSS |
| Salsal IGHJ3D*01 F | HAFDYWGKGTMVTVSS |
| Salsal IGHJ4D*01 F | YGFDYWGKGTMVTVSS |
| Salsal IGHJ5D*01 F | YAAFDYWGQGTIVTVSL |
